# Anesthetic management of a patient with sickle β^+^ thalassemia

**DOI:** 10.4103/1658-354X.76496

**Published:** 2011

**Authors:** Saswata Bharati, Subhabrata Das, Prasenjit Majee, Subrata Mandal

**Affiliations:** *Department of Anesthesiology, Midnapore Medical College, West Bengal, India*; 1 *Department of Surgery, Midnapore Medical College, West Bengal, India*

**Keywords:** *Cholecystectomy*, *sickle cell disease*, *sickle β^+^ thalassemia*, *sickle β^0^ thalassemia*, *thalassemia*

## Abstract

Sickle cell disease is a congenital condition and its most common clinical manifestation is anemia due to chronic hemolysis. Persistent and accelerated hemolysis associated with multiple transfusions is a recognized risk factor for the development of cholelithiasis. The occurrence of gallstones is one of the most important manifestations of sickle cell disease in the digestive tract. Most gallstones are pigmented and characteristically occur at younger ages and the prevalence of cholelithiasis increases progressively with age, affecting 50% of young adults. Cholecystectomy is the most common surgical procedure performed in sickle cell disease patients. Anesthesia in this population of patients for major surgeries deserves special attention due to various complications particularly silent infarctions of end organs are common. We are reporting a 14-year-old girl diagnosed with sickle cell anemia and β^+^ thalassemia with cholelithiasis went for cholecystectomy under general anesthesia. Although the patient has both β^+^ thalassemia and sickle cell disease component, the latter is of more concern for anesthesia.

## INTRODUCTION

Sickle cell disease (SCD) is the most commonly found hemoglobinopathy. It can be combined with thalassemias or hemoglobin C variety producing more complicated blood picture. Advances in therapeutic techniques and anesthetic procedures have led to a considerable increase in the success of surgical procedures in these patients. We report the case of a 14-year-old girl diagnosed with sickle cell anemia and β^+^ thalassemia who presented with chronic cholecystitis. She was scheduled for cholecystectomy.

## CASE REPORT

A 14-year-old female patient was admitted to the surgical department of Midnapore Medical College with chief complaints of recurrent right hypochondrium pain and recurrent episodes of jaundice. At the age of 7 years she developed jaundice for the first time which was gradual in onset. She felt pain at right hypochondrium 3 years back for the first time which was associated with fever and vomiting. She was treated conservatively for the initial episodes but the severity gradually deepened. She had no blood transfusion before. Her built was thin and when examined was found to have mild pallor, moderate icterus and “hemolytic facies” characterized by frontal bossing, intercanthal widening, depressed bridge of nose, malar prominence, and muddy sclera. On examination, right hypochondrium area was to be found tender without any obvious lump formation. No organomegaly was detected. Examination of other systems did not reveal any other abnormality. Ultrasonographic finding revealed contracted gallbladder with multiple echogenic calculi within the gallbladder lumen. Blood investigation showed total Hb as 10.7 g/dL. The reports of Hb variant analysis and liver function tests are shown in Tables 1 and 2, respectively. The diagnosis was made as sickle β^+^ thalassemia with gall stones and the patient was advised open cholecystectomy on anticipation of high degree of adhesions. She was transfused with 1 U of packed red blood cell (RBC), after which the hemoglobin became 11.6 g/dL. The post-transfusion Hb variant analysis is shown in Table 1.

**Table 1:** Hemoglobin variant analysis of blood (HPLC technique)

Investigation	Observed value (%)	
	Pre transfusion	Post transfusion
Hb A	3.9	20.2
Hb A2	2.4	2.7
Hb F	20.6	17.9
Hb S	70.2	58.9
Hb D	Absent	Absent
Hb C	Absent	Absent

**Table 2:** Liver function test

Investigation	Observed value
Total bilirubin	4.8 mg/dL
Unconjugated bilirubin	3.1 mg/dL
SGPT	76 U/L
SGOT	65 U/L
Total protein	7.3 g/dL
Albumin	3.0 g/dL

The patient was explained with the nature of operation and anesthesia and an informed consent was taken. She was given alprazolam 0.25 mg as preoperative medication at the night before the operation. To maintain and monitor adequate hydration the patient was catheterized on the night before surgery. She was stopped from taking oral fluids 4 h prior to surgery while Ringer’s lactated solution was started to maintain hydration through intravascular catheter. The target urine output was maintained over 1 ml/kg/h. Injection ondansetron 100 µg/kg, fentanyl 2 µg/kg, and midazolam 2 mg were given intravenously before induction. She was intubated with 7.0 size cuffed endotracheal tube orally after inducing with propofol (2 mg/kg) and rocuronium (1.2 mg/kg). The anesthesia was maintained with isoflurane, and intermittent dose of fentanyl. Nitrous oxide was avoided. During intraoperative period standard monitoring was done with non -invasive blood pressure (NIBP), electrocardiogram (ECG), pulse oximetry (SpO_2_), end tidal CO_2_(EtCO_2_). Postoperatively the patient was shifted to high dependence unit.

## DISCUSSION

The sickle cell syndromes are caused by a genetic mutation in the β-globin chain of hemoglobin molecule, where glutamic acid is substituted by valine at the sixth position and include the prototype sickle cell anemia (homozygous), sickle cell trait (heterozygous), hemoglobin SC disease, and sickle β thalassemia (S/β° thalassemia and S/β^+^ thalassemia). Patients with these disorders commonly suffer a multitude of destructive events to vital organs, especially to the central nervous system, spleen, bones, kidneys, lungs, and heart as a result of microvascular plugging by the sickled erythrocytes. The erythrocytes undergo sickling when they are deoxygenated in the presence of hypoxia, resulting into decreased solubility and reversible polymerization of sickle hemoglobin (HbS), thereby increasing the intracellular viscosity and deforming the shape of the cells. These abnormalities produce microvascular vaso-occlusion and premature red cell destruction. High level of HbS (homozygous variety), dehydration, infection, acidosis, and hypothermia are the factors which make the erythrocytes susceptible for sickling. General anesthesia and surgical trauma add additional risk of complications because of changes in temperature, pH, oxygen tension, and fluid volume. Circulatory stasis and suboptimal ventilation during surgery allow HbS to polymerize in the capillaries with subsequent ischemic infarcts in many tissues. The reported morbidity for SCD patients having major operations approaches 40%.[1]

A significant number of patients develop calcium bilirubinate cholelithiasis due to hemolysis and possibly cholecystitis as a result of the continual increased load of bile salts resulting from the shortened lifespan of the cells containing HbS. Gallstones are found in about 30 to 50% of children with sickle cell anemia.[2] By adulthood, 50 -70% of sickle cell patients have gallstones.[3,4] About 50% of these patients with gallstones may be asymptomatic.[2] Elective cholecystectomy is indicated for those who are symptomatic, but, because of operative mortality, there is disagreement concerning surgery for asymptomatic patients.[5–7]

Several sickle diseases occur as the result of inheritance of HbS from one parent and another hemoglobinopathy, such as β thalassemia or HbC, from the other parent. Sickle cell syndrome can be associated with β thalassemia in two ways: S/β° thalassemia and S/β^+^ thalassemia. In S/β° thalassemia hemoglobin level remains within 7-10 g/dL and is characterized by vaso-occlusive crises; aseptic necrosis of bone. S/β^+^ thalassemia is characterized by 10-14 g/dL hemoglobin, but the incidence of vaso-occlusive crises and bone necrosis are rare. The small amount of HbA present in S/β^+^ thalassemia tends to minimize the complications caused by the sickled cells. The values of different types of hemoglobin variants are described in Table 3.

**Table 3:** Hemoglobin variants in S/β^+^ thalassemia and S/β° thalassemia

Investigation	S/β^+^ thalassemia (Double Heterozygote)	S/β° thalassemia (Double Heterozygote)
HbS	Typically 60-80%	75-90%
HbA1	15-30%	Absent unless transfused
HbA2	3-8%	5-8%
HbF	2-20%, higher for age in infants	5-20% in adults, higher for age in infants

Modern transfusion therapy, consisting of multiple small transfusions or exchange transfusion of HbA erythrocytes administered over several weeks prior to the operation, not only corrects the chronic anemia and thereby increases the oxygen carrying capacity but suppresses erythropoiesis of cells containing HbS in the patient’s bone marrow. However, increasing the hemoglobin level to over 11 g/dL increases blood viscosity and may thus cause complications. Also, these patients are well tolerated to the low (6-9 g/dL) Hb level, partly because of a low oxygen affinity of HbS within the RBC resulting into marked shifting of the oxygen dissociation curve. Simple top-up transfusions are given when the Hb level is < 9 g/dl and exchange transfusion is reserved for patients with higher Hb value with very high HbS value to prevent rise in blood viscosity. Recent randomized trials have shown that the conservative transfusion regimen (i.e. simple top-up transfusion to bring the hemoglobin level up-to 11 g/dL, irrespective of HbS concentration) is as effective as an aggressive one (i.e. exchange transfusion with aim to reduce the HbS to < 35%) in decreasing perioperative complications in SCD patients and is associated with only half as many transfusion-associated complications.[8,9]

An increasingly recognized complication of SCD and other hemolytic anemia is pulmonary artery hypertension (PAH), now recognized as a major mortality risk factor in adults and affecting approximately 20-40% of individuals with SCD.[10,11] The mechanism of PAH in SCD is thought to result from the effect of hemolysis on nitric oxide pathways leading to impaired vascular endothelial function and abnormal vascular tone.[12] The incidence of pulmonary hypertension in patients with HbS/β -thalassemia is similar to that observed in patients with SCD.[13] Therefore, nitrous oxide should preferably be avoided in fear of further aggravating the pulmonary hypertension.

The patient must be free of any acute illness, especially one involving the respiratory system. Adequate hydration preoperatively combined with avoidance of perioperative hypoxia, hypothermia, and acidosis, the triggers for sickling, will reduce the sickle-induced complications.[14–16] Basic supportive care, including adequate analgesia, early mobilization, and oxygen supplementation to prevent hypoxemia, is the mainstay of postoperative management.
